# Design and Synthesis Optimization of Fluorescent Acrylate-Based and Silicate-Based Materials for Carbonyl Adsorption

**DOI:** 10.3390/polym17131843

**Published:** 2025-06-30

**Authors:** Laura Carballido, Thomas Karbowiak, Elias Bou-Maroun

**Affiliations:** Institut Agro, Université Bourgogne Europe, INRAE, UMR PAM, F-21000 Dijon, France; laura.carballido@agrosupdijon.fr

**Keywords:** acrylates, sol-gel, fluorescence, sensor, lipid oxidation

## Abstract

For their use as chemical sensors, the optimization of the performance of polymeric materials is a critical step in their development for the desired application. The main objective of this work was to identify the best-suited materials to develop a sensor for carbonyl monitoring based on fluorescence. Two categories of materials were compared: acrylate-based materials, obtained by radical polymerization, and silicate-based materials, obtained by sol-gel synthesis. The performances of these materials in terms of yield of polymerization, carbonyl adsorption capacity and fluorescence property were compared. More precisely, the influence of various synthesis parameters such as polymerization type, radical polymerization initiation method, the nature of the functional monomer and the molar ratio of the different reactants was assessed. On the first hand, among acrylate-based materials, the one based on 4-vinylaniline showed better adsorption capacity compared to those based on 3-vinylaniline and 2-vinylaniline. Moreover, materials obtained by UV-polymerization showed a better adsorption capacity compared to those obtained by thermally initiated polymerization. On the other hand, the silicate-based material provided a better synthesis reproducibility, a higher adsorption capacity and a higher fluorescence intensity compared to its acrylate-based counterparts. Finally, contrary to the acrylate-based materials tested, the adsorption capacity and fluorescence properties of silicate-based materials were stable over time.

## 1. Introduction

The monitoring of carbonyl compounds is of high interest in many fields. They can provide information about the level of air pollution [1], be used as food quality trackers [2] or even as biomarkers in medical diagnoses [3]. Classical analytical methods used to determine the amount of carbonyl compounds in complex matrices often rely on chromatography [4]. Although this technique is very efficient, it needs expensive equipment operated by highly trained personnel, as well as time-consuming sample preparation and analysis. This underscores the demand for simpler yet still sensitive and accurate analytical procedures. Optical sensors, whether they are colorimetric, fluorometric or sometimes chemiluminescent, are gaining increasing interest for the development of new user-friendly analytical methods [5].

Chemical sensors for carbonyl monitoring could rely on acrylate-based or silicate-based polymers. Acrylate-based materials are organic polymers, usually obtained by radical polymerization. This synthesis route requires the use of one or more functional monomers, a cross-linker, a polymerization initiator and a solvent [6]. Functional monomers must be carefully chosen according to the application. They must possess functional groups providing the targeted reactivity to the resulting polymer. The most widely used monomers are acrylamides and vinyl esters. The cross-linker confers mechanical stability to the final material. The most used cross-linkers are divinylbenzene and ethylene glycol dimethacrylate (EGDMA). Radical polymerization is usually performed either thermally or photo-initiated. 2,2′-azobis (2-methylpropionitrile) (AIBN) is classically used as a thermal initiator and 2,2-dimethoxy-1,2-diphenylethanone (DMPAP) is commonly used as a photo-initiator. As an example, Rico-Yuste et al. already studied the synthesis of an acrylate-based material for the colorimetric detection of furfural in beers [7]. The sensing material was obtained by the co-polymerization of two functional monomers: 4-vinylaniline and 2-hydroxymethyl methacrylate, using EGDMA as a cross-linker.

Silicate-based materials are usually made of a mineral structure on which organic parts can be grafted. One way to prepare such materials is the sol-gel process. It relies on the hydrolysis and condensation of alkoxysilane derivatives under acidic or basic conditions [8]. The co-condensation of an organosilicate monomer with a tetraalkoxysilane cross-linker, such as tetraethoxysilane (TEOS), provides tunable properties to the resulting material. Silicate-based materials show better chemical and physical stability compared to their acrylate-based counterparts. Moreover, no organic solvents and low toxicity reactants are used for the sol-gel synthesis [9], making them interesting from the point of view of green chemistry. Since the development of mesoporous silica for catalytic and adsorption applications [10], silicate-based materials have been extensively functionalized, but not used as a fluorescent sensor for carbonyl monitoring. For the fluorescent detection of primary amines, Seok et al. recently proposed a sol-gel material functionalized with styrylbenzene derivatives and aldehyde groups [11]. The objective of the project presented in this work is based on the opposite concept: to develop a fluorescent sensor containing amine groups to monitor carbonyl compounds.

This paper reports on the design and synthesis of acrylate-based and silicate-based materials intended to be used as chemical sensors for carbonyl monitoring. The first objective of the present work was thus to evaluate the best candidates for such a purpose, comparing the more traditional materials based on acrylates to the less explored sol-gel materials. The synthesis parameters strongly influence the performance of the final materials. However, only a few studies detailed the synthesis optimization process and the impact of selected parameters on the properties of the materials. The second objective was therefore to study the influence of the most significant parameters on the materials’ performance: the polymerization route, the radical polymerization initiation method, the nature of the functional monomer and the molar ratio of the different reactants. The resulting materials were compared in terms of hexanal adsorption capacity and fluorescence properties.

## 2. Materials and Methods

### 2.1. Chemicals

4-vinylaniline (4-VA, 97%, CAS number 1520-21-4), 3-vinylaniline (3-VA, 97%, CAS number 15411-43-5), ethylene glycol dimethacrylate (EGDMA, 98%, CAS number 97-90-5), tetraethylorthosilicate (TEOS, 99%, CAS number 78-10-4), 2,2-dimethoxy-1,2-diphenylethanone (DMPAP, 99%, CAS number 24650-42-8), 2,2′-azobis (2-methylpropionitrile) (AIBN, 98%, CAS number 78-67-1), octan-1-ol (98%, CAS number 111-87-5) ammonium hydroxide (NH_4_OH, 30%, CAS number 1336-21-6) and ethanol for UV-spectroscopy Uvasol^®^ (EtOH, 99,9%, CAS number 64-17-5) were purchased from Sigma Aldrich, Saint Quentin Fallavier, France. 2-vinylaniline (2-VA, 95%, CAS number 3867-18-3) and 4-trimethoxysilylaniline (4-TMSA, 97%, CAS number 33976-43-1) were purchased from abcr, Karlsruhe, Germany. Acetonitrile (CH_3_CN, 99.9%, CAS number 75-05-8) was obtained from Honeywell, Charlotte, NC, USA. Hexanal (98%, CAS number 66-25-1) was purchased from Alfa Aesar, Paris, France. Deionized water was used, obtained from a MilliQ RG, Millipore apparatus, Burlington, MA, USA.

### 2.2. Materials Synthesis

Only the final experimental procedures with optimized parameters are reported in the following. For the synthesis of acrylate-based materials, the initiator and solvent ratios were optimized. For silicate-based materials, the temperature of reaction and the volume of solvent were investigated.

Acrylate-based materials

The functional monomer (4-vinylaniline, 3-vinylaniline or 2-vinylaniline) was first dissolved in acetonitrile. The cross-linker ethylene glycol dimethacrylate (EGDMA) and the initiator (either DMPAP or AIBN) were then added. The following molar ratio was used: 4/20/1/50 for the functional monomer/cross-linker/initiator/solvent. Oxygen was removed from the reaction mixture by nitrogen bubbling for 10 min. For photoinitiated polymerization, the reaction medium was placed at 10 cm from an UV lamp (366 nm) for 24 h. For thermally initiated polymerization, the reaction mixture was stirred at 250 rpm and heated at 60 °C for 24 h. The resulting powder was washed 3 times with acetonitrile by Büchner filtration using a 0.2 µm pore membrane. The material was dried 12 h at 60 °C, crushed and stored at 4 °C in the dark before use. A material with a higher ratio of functional monomer (20/20/1/50) was also synthesized following the same procedure. The polymerization yield was calculated using the theoretical mass of material supposedly obtained.

Silicate-based materials

4-trimethoxysilylaniline (4-TMSA) was first dissolved in an ethanol-in-water mixture (1/10 molar ratio) at 60 °C for 10 min. Tetraethyl orthosilicate (TEOS) and ammonium hydroxide (30% NH_4_OH) were then added. The following molar ratio was used: 1/5/31.25/250/25 for 4-TMSA/TEOS/NH_4_OH/H_2_O/EtOH. The mixture was stirred at 500 rpm for 24 h at 22 °C. Then, the solvent was evaporated under the hood, and the resulting powder was washed 3 times with an ethanol in water mixture (1/10 molar ratio) by Büchner filtration using a 0.2 µm pore membrane. The material was dried for 12 h at 60 °C, crushed and stored at 4 °C in the dark before use. A material with a higher ratio of functional monomer (5/5/31.25/250/25) was synthesized following the same procedure. The polymerization yield was calculated from the assumed theoretical mass of material obtained, considering the loss of alcohol and water molecules during the hydrolysis and condensation processes.

In both cases (acrylate or silicate-based materials), control materials were systematically synthesized following the same procedure but without functional monomer. This aims at proving that the adsorption of carbonyl compounds and subsequent material fluorescence were strictly due to the presence of the monomer present in the structure of the material.

The materials were named according to the nature of their functional monomer, the polymerization route, the initiation of the polymerization and the molar ratio of functional monomer. The following table (Table 1) summarizes the names of the materials studied in this work:

### 2.3. Materials Characterization

Fourier Transform Infrared Spectroscopy (FTIR)

FTIR absorbance spectra of the materials and their corresponding blank (obtained without functional monomer) were recorded at 25 °C using a Nicolet iS10 (Thermo Scientific^TM^, Waltham, MA, USA) spectrometer. Before analysis, around 1 mg of dried powder material was dispersed into KBr to obtain pellets of 200 mg. The transmission spectra were recorded in a spectral range from 400 to 4000 cm^−1^ with a resolution of 4 cm^−1^ and 16 scans per analysis, using a 200 mg KBr pellet as the reference.

Scanning electron microscopy (SEM)

The surface morphology of the materials was characterized by SEM using a JEOL 6700F microscope equipped with a cold FEG electron source operating at 1 kV and an Everhart–Thornley detector combined with an in-lens. Prior to imaging, the powder was deposited on a carbon tape and the surface of the material was coated with carbon. Images were acquired at various magnifications between 100 and 20,000.

Laser diffraction

The particle size distribution was determined by laser diffraction using a Malvern MasterSizer 3000 (Malvern Instruments Ltd., Malvern, Worcestershire, UK). The material was added to the circulating water until the obscuration reached 8% to 10%. Stirring speed was set to 1200 rpm. The temperature was maintained at 22 °C throughout the experiment. For the acrylate-based materials, the refractive index of the particles (1.488) was estimated using the polymethyl methacrylate model. For the silicate-based material, the refractive index of the particles (1.457) was based on the silica model [12]. The refractive index of the dispersing phase was set at 1.36. The Mie diffusion model (r ~ λ) was used to calculate particle size based on the following hypotheses: monochromatic incident light, spherical particles, complex refractive index and isotropic surrounding medium. The accuracy of the equipment is 0.5% and repeatability is 0.6%. The wavelength used is 632 nm. Each measurement was performed 3 times.

Material toxicity assessment

The mutagenicity of the material was estimated by the Ames’ test, performed on two bacterial strains: *Salmonella typhimurium TA98 and TA100*, according to Maron and Ames [13] and the OECD guideline no. 471 for the testing of chemicals [14]. More details about the experimental procedure can be found in previous work [9].

### 2.4. Adsorption Capacity of the Materials

The adsorption capacity of hexanal by the materials was determined by gas chromatography. A total of 5 mg of material was suspended in 2 mL of hexanal (2.37 × 10^−3^ mol·L^−1^) in acetonitrile solution. Octan-1-ol (2.54 × 10^−3^ mol·L^−1^) was added as an internal standard. Samples were agitated at 50 rpm for 24 h at 22 °C. They were centrifuged at 10,000 rpm for 10 min. The remaining concentration of hexanal in the liquid phase was determined by injecting 1 μL of the supernatant using an autosampler (Gerstel, Mülheim an der Ruhr, Germany) on a 7890 A gas chromatograph (Agilent Technologies, Santa Clara, CA, USA) equipped with a capillary column (30 m × 0.32 mm internal diameter, 0.5 μm thickness) coated with a DB-5MS stationary phase (Agilent J&W). Injections were performed in split mode (split ratio 50:1). Injection temperature was 250 °C. Helium was used as carrier gas at a flow rate of 1.4 mL·min^−1^. A mass spectrometer 5975C (Agilent) was used as the detector (electronic impact 70 eV, chemical ionization source at 230 °C, transmission line at 240 °C). The oven temperature was programmed from 50 °C to 65 °C at a rate of 3 °C·min^−1^, from 65 °C to 115 °C at 30 °C·min^−1^, then from 115 °C to 140 °C at 3 °C·min^−1^ and from 140 °C to 250 °C at 30 °C·min^−1^. The oven temperature was finally maintained at 250 °C for 5 min. A solvent delay of 3 min was also applied. Each adsorption experiment was carried out 3 times.

The adsorption capacity is defined as the mass of hexanal adsorbed by 1 g of material. It was calculated as follows:

(1) $$Adsorption\;capacity=\frac{\left(Ci-Cf\right)\times V_{liq.}}{m}$$

with *Ci*, the initial concentration of hexanal in the liquid phase in contact with the material, *Cf*, the concentration of hexanal remaining in the liquid phase at equilibrium, *V_liq._*, the volume of the hexanal solution, and *m*, the mass of the material.

Statistical analysis (ANOVA with Tukey’s post hoc analysis) was performed using XLSTAT (Addinsoft, Paris, France) software. Significance was established at *p* < 0.05.

### 2.5. Fluorescence of the Materials

The fluorescence emission spectrum of the materials was recorded before and after exposure to hexanal in ethanol. A total of 10 mg of material was suspended in 2 mL of ethanol. After 10 s of stirring, the fluorescence emission spectrum of the suspended material was recorded. Then, 500 μL of a solution of hexanal was added to obtain a concentration in the measurement cell of 9.98 × 10^−3^ mol·L^−1^. After 15 min of stirring at 80 rpm, the fluorescence emission spectrum of the material was recorded again. The fluorescence intensity of the material after exposure to hexanal was compared to its initial fluorescence. A Spectrofluorometer Fluoromax4^®^ (Horiba, Kyoto, Japan) was used with the software Fluoressence^®^. A SUPRASIL^®^ quartz cell (Hellma, Müllheim, Germany) of a 10 × 10 mm dimension was employed. The excitation wavelengths were 280 ± 2 nm for acrylate-based materials and 290 ± 2 nm for silicate-based materials. Fluorescence emission spectra were recorded between 300 nm and 500 nm, with a 2 nm bandpass and a step interval of 1 nm. The temperature was kept at 22 °C during the experiment. Each experiment was performed 3 times.

The percentage of fluorescence quenching, *Q*, was determined as follows:

(2) $$Q=\frac{I_{0}-I_{eq}}{I_{0}}\times 100$$

with *I*_0_, the intensity of fluorescence at 337 nm of the material in ethanol before the addition of carbonyl and *I_eq_*, its intensity after exposure to carbonyl at a concentration of 9.98 × 10^−3^ mol·L^−1^ for 20 min.

## 3. Results and Discussion

### 3.1. Design of the Materials

The objective of this work was to develop new materials for carbonyl sensing based on fluorescence. The tested synthesis routes and reactants were carefully selected to primarily meet the following requirements: a synthesis easy to implement and functional monomers commercially available at a reasonable cost. Moreover, to respect the principle of green chemistry related to the use of safe reactants, only chemicals with limited toxicity were chosen. This would enable the final use of the resulting sensor in fields requiring safe sensors, such as food, cosmetics or medicine.

Two main synthesis routes were considered. The first one was the radical polymerization of acrylate-based monomers. The second one was the sol-gel polymerization of silicate-based reactants.

To optimize the detection, a single functional monomer combining the two required properties was sought: it should be able to interact with carbonyl compounds and be fluorescent. Aniline-based monomers are perfectly suited for that purpose, as the primary amine group can react with carbonyls and the aniline is a fluorescent core. Acrylate-based as well as silicate-based monomers with aniline cores were commercially purchased. The commercial availability of acrylate-based monomers is greater than that of silicate-based monomers. Three monomers were selected for radical polymerization (4-vinylaniline, 3-vinylaniline, 2-vinylaniline) and a single monomer for the sol-gel synthesis (4-trimethoxysylilaniline).

The adsorption capacities towards hexanal and the subsequent change in the fluorescence properties of the resulting sensing materials were investigated to identify the best candidate to be used as a sensor for carbonyl monitoring.

### 3.2. Synthesis Optimisation

As a first step, synthesis parameters were optimized to maximize the yield of polymerization.

- Acrylate-based materials

Acrylate-based materials were synthesized by radical polymerization by adapting a protocol from previous work [15]. The following parameters were studied for optimization:

- Initiation of polymerization: UV-light or heat (60 °C)
- Nature of the functional monomer: 4-vinylaniline, 3-vinylaniline, 2-vinylaniline
- Functional monomer/initiator molar ratio: 4/0.5 or 4/1
- Functional monomer/solvent molar ratio: 4/100 or 4/50.

**Figure 1:**
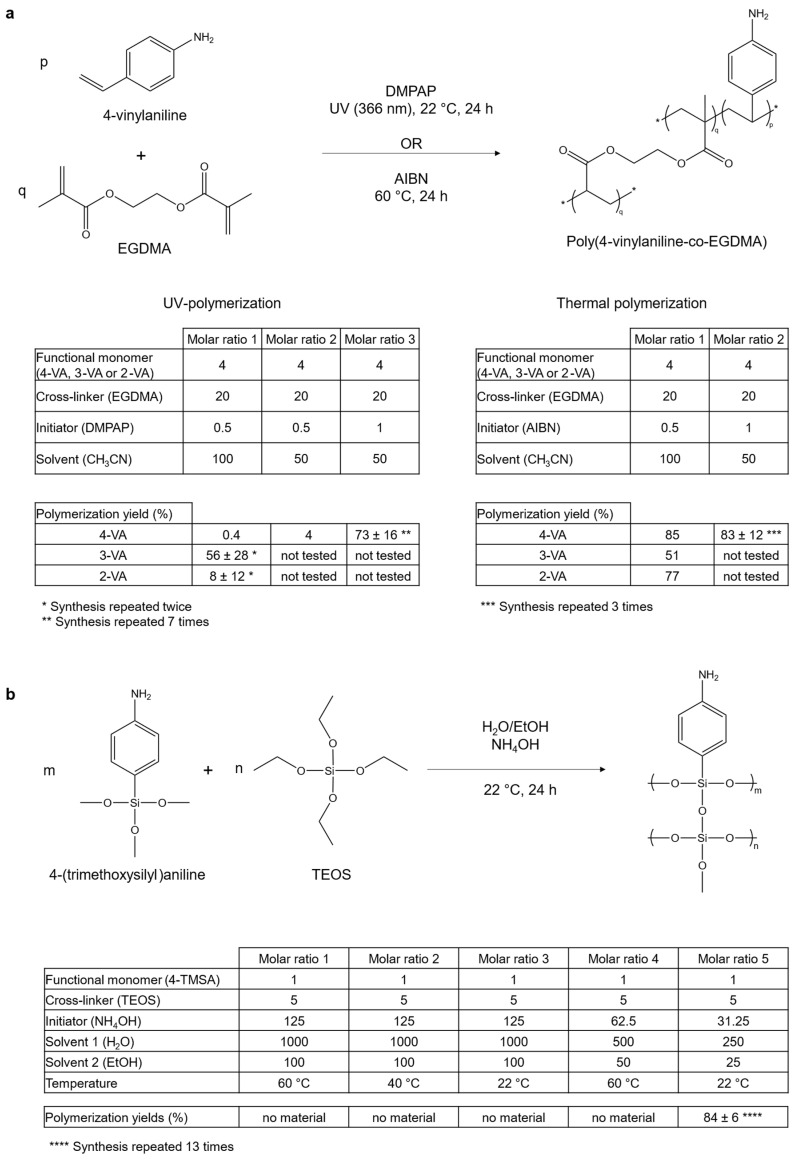
Optimization of the synthesis with investigated parameters. (**a**) Reaction route and molar ratios used for the synthesis of acrylate-based materials using 4-vinylaniline (4-VA) as a functional monomer. Similar reactions were performed with 3-vinylaniline (3-VA) and 2-vinylaniline (2-VA) for the molar ratio of 1. Radical polymerization was either photoinitiated or thermally initiated. When no standard deviations for the yield of polymerization are given, the synthesis was performed only once. p and q are the molar ratios of functional monomer and cross-linker used for the synthesis of the acrylate-based materials, respectively. m and n are the molar ratios of functional monomer and cross-linker used for the synthesis of the functionalized sol-gel material, respectively. (**b**) Reaction route and molar ratios used for the synthesis of silicate-based material.

Figure 1a summarizes the reaction route for the acrylate-based materials. It also gives the yields of polymerization obtained according to the different tested parameters.

Firstly, considering UV-polymerization, a two-fold decrease in the volume of solvent led to improved yield of polymerization—from 0.4% to 4%—using 4-VA monomer. As shown in a previous work [16], a reduced amount of solvent favors the precipitation of polymers from the reaction mixture. A change in the solvent nature was also tested by replacing acetonitrile with methanol. This positively affected the yield of polymerization, which was 59% using 4-VA monomer. But this material was no longer considered, as alternative strategies of polymerization gave similar or even better yields using less toxic solvents (acetonitrile and ethanol/water mixtures). A two-fold increase in the quantity of initiator also improved the yield of polymerization, from 4% to 73%, using 4-VA monomer. It could be explained by the residual amount of oxygen remaining in the reaction mixture, which can combine with free radicals generated during the first step of the radical polymerization and thus inhibit polymerization. Oxygen is supposedly removed before polymerization, but it is possible that the nitrogen bubbling used for this purpose was not effective enough. A higher amount of initiator could thus compensate for the radicals inhibited by oxygen. The same hypothesis was considered to explain the high variability observed for the yield of reaction when the same synthesis was repeated many times (73 ± 16% for 4-VA UV on seven syntheses). Increasing even more the molar ratio of the initiator could probably solve this minor issue.

Secondly, for thermally initiated polymerization, neither the initiator ratio nor the solvent volume significantly impacted the yield of polymerization, contrary to UV-polymerization. The best yields of polymerization were obtained when temperature (60 °C) was used to initiate the polymerization instead of UV-light (83% and 73%, with 4-VA, respectively). This was attributed to the better control of the temperature homogeneity in the reaction mixture for thermally initiated polymerization. In the case of UV-polymerization, only a single side of the reaction tube was exposed to the UV light, therefore limiting the opportunities for starting new polymeric chains in the whole reaction mixture.

b. Silicate-based materials

Silicate-based materials were synthesized using a sol-gel process. The temperature and the solvent molar ratio were optimized to favor the precipitation of the material.

Figure 1b displays the reaction route for the synthesis of silicate-based materials. The yields of polymerization according to the different parameters investigated are also reported. It was impossible to synthesize any material when only the temperature of the reaction was modified. It was only when the solvent ratio was reduced by four that the material finally precipitated. Compared to acrylate-based materials, the synthesis repeatability was better controlled (83 ± 8% for 4-TMSA sol-gel and 73 ± 16% for 4-VA UV, based on 7 syntheses in each case). This is likely because, unlike radical polymerization, sol-gel polymerization is not affected by the presence of oxygen.

To conclude on that first optimization step, when dealing with radical polymerization, an important parameter to control in order to increase the yield of reaction appears to be related to the quantity of residual oxygen dissolved in the reaction mixture. The molar ratio of the initiator should therefore be chosen accordingly. The solvent volume should also be limited to favor the material precipitation. This last consideration applies to all types of considered polymerization. Based on the yield of polymerization and the reproducibility of the synthesis, the classification between the different synthesis routes would follow: sol-gel polymerization > thermal polymerization > UV-polymerization.

### 3.3. Adsorption Capacity of the Materials

**Figure 2:**
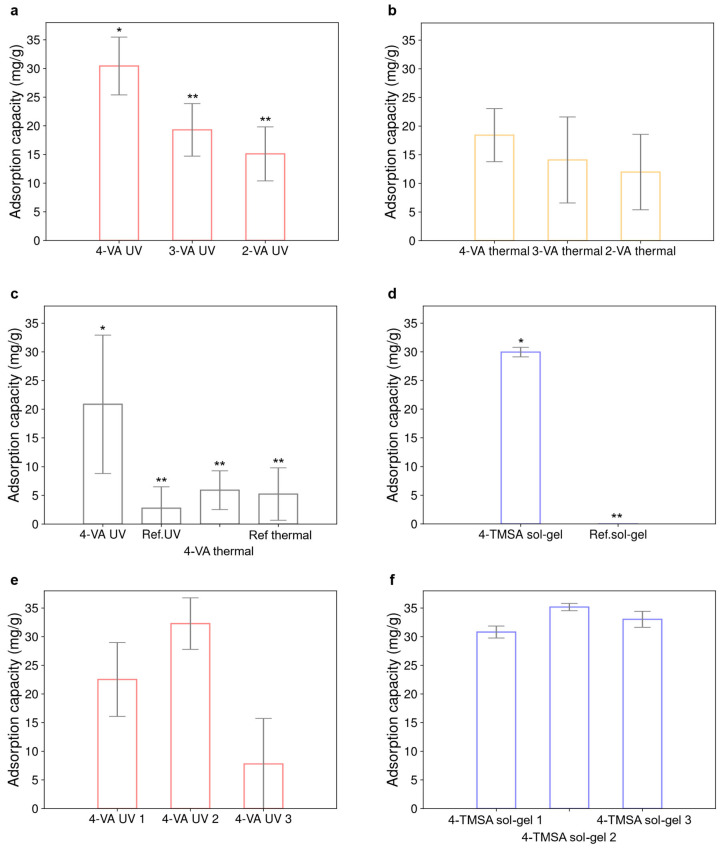
Adsorption capacity of the materials, reported in mg of hexanal adsorbed by g of material in acetonitrile. Initial concentration of hexanal was 2.37 × 10^−3^ mol·L^−1^. (**a**) Adsorption capacity of the materials obtained by UV-polymerization. The following molar ratio was used: 4/20/0.5/100 for the functional monomer/cross-linker/initiator/solvent. (**b**) Compared adsorption capacity of materials obtained by polymerization, thermally initiated. The following molar ratio was used: 4/20/0.5/100 for the functional monomer/cross-linker/initiator/solvent. (**c**) Impact of the polymerization initiation on the adsorption capacity of the acrylate-based materials. The following molar ratio was used: 4/20/1/50 for the functional monomer/cross-linker/initiator/solvent. (**d**) Adsorption capacity of the sol-gel material and its reference made without functional monomer. (**e**) Adsorption capacity of the 4-VA UV material obtained from 3 distinct synthesis batches. The following molar ratio was used: 4/20/1/50 for the functional monomer/cross-linker/initiator/solvent. (**f**) Adsorption capacity of the 4-TMSA sol-gel material obtained from 3 distinct synthesis batches. In each case, the error bar represents the standard deviation obtained for three replicates. Statistical comparisons were performed using one-way ANOVA test. Significantly different values (*p* < 0.05) are indicated by different number of stars.

Since the synthesized materials were intended to be used as carbonyl sensors, their hexanal adsorption capacity was systematically assessed. Figure 2 depicts the adsorption capacity of the different materials exposed to a single concentration of hexanal in acetonitrile.

Figure 2a,b compares the influence of the nature of the functional monomer on the adsorption capacity of the resulting materials. Figure 2a focuses on the materials obtained by UV-polymerization. The material synthesized from the 4-vinylaniline monomer adsorbs a higher quantity of hexanal (30 ± 5 mg·g^−1^) compared to materials obtained with 3-vinylaniline and 2-vinylaniline (19 ± 5 mg·g^−1^ and 15 ± 5 mg·g^−1^, respectively). The decrease in adsorption capacity can be attributed to the increasing steric effects depending on the position of the substituent groups on the aniline ring. The meta- and the ortho-substitutions may lead to a reduced accessibility of hexanal to the primary amine function compared to the para-substitution. This is probably due to a closer proximity of the adsorption site to the polymeric chain. When radical polymerization was thermally initiated, a similar behavior was observed for the three materials (Figure 2b). Only the material obtained by UV-polymerization with the 4-vinylaniline adsorbed a significantly higher quantity of hexanal compared to the five other materials shown in Figure 2a,b. Figure 2c compares the impact of the polymerization initiation route on the adsorption capacity of the materials synthesized using the 4-vinylaniline monomer. The material synthesized by UV-polymerization might adsorb about three times more compared to the material polymerized with temperature (20 ± 12 mg·g^−1^ and 6 ± 3 mg·g^−1^, respectively). This could be explained by a difference in the distribution of the aniline groups at the surface of the material. With thermal initiation, adsorption sites could be entrapped within the bulk structure, thus being less accessible to hexanal. Piletska et. al. already suggested that photoinitiated polymerization produced materials with better performances [16]. Reference materials were systematically synthesized following the same experimental procedure but without using any functional monomer. The adsorption capacity of the 4-VA UV material significantly differs from the adsorption capacity of its reference. However, this is not the case for the material obtained by thermal initiation. It can also be noticed that both references could adsorb a small quantity of hexanal (3 ± 3 mg·g^−1^ for the UV reference material and 5 ± 4 mg·g^−1^ for the thermal reference material). This could be attributed to hydrogen bonding formed between hexanal and the ethylene glycol dimethacrylate cross-linker. Figure 2a,b display the adsorption capacity of materials synthesized with a functional monomer/cross-linker/initiator/solvent molar ratio of 4/20/0.5/100 while materials reported in Figure 2c were obtained with a functional monomer/cross-linker/initiator/solvent molar ratio of 4/20/1/50. Figure 2d shows the adsorption capacity of the sol-gel material (29 ± 1 mg·g^−1^), which significantly differs from that of its reference. The standard deviation is drastically reduced using this synthesis route compared to the radical polymerization. This is in favor of a more homogeneous material, as already reported in the literature [17]. Figure 2e,f compare the reproducibility of the performance of the materials when their synthesis was repeated several times. Figure 2e compares the adsorption capacity of the 4-VA material obtained by UV-polymerization for three distinct synthesis batches. The three materials show a significant difference in their adsorption capacity (coefficient of variation of 58%). In contrast to that, the sol-gel material displayed only a very low variability (3%) (Figure 2f). Therefore, it seems more difficult to achieve synthesis reproducibility with radical polymerization than with sol-gel polymerization.

To conclude, 4-vinylaniline appeared to be the monomer providing the highest adsorption capacity to the material compared to 3-vinylaniline and 2-vinylaniline. A better adsorption capacity was obtained for materials synthesized by UV-polymerization compared to those resulting from thermal polymerization. The sol-gel material showed both better adsorption capacity and higher synthesis reproducibility.

According to their adsorption capacity, the different materials could be classified in the following order: 4-TMSA sol-gel material > 4-VA UV > 4-VA thermal > 3-VA UV > 2-VA UV > 3-VA thermal > 2-VA thermal. For acrylate-based materials, those obtained using 3-vinylaniline and 2-vinylaniline, as well as those resulting from thermal polymerization, were no longer considered for further experiments since their adsorption capacities were lower than that of the 4-VA UV material and also more expensive to produce. Only the 4-VA UV and the 4-TMSA sol-gel materials were thus further investigated.

### 3.4. Fluorescence Properties of the Materials

To use the synthesized materials as fluorescent sensors for carbonyl detection, their fluorescence properties after interaction with hexanal were investigated.

**Figure 3:**
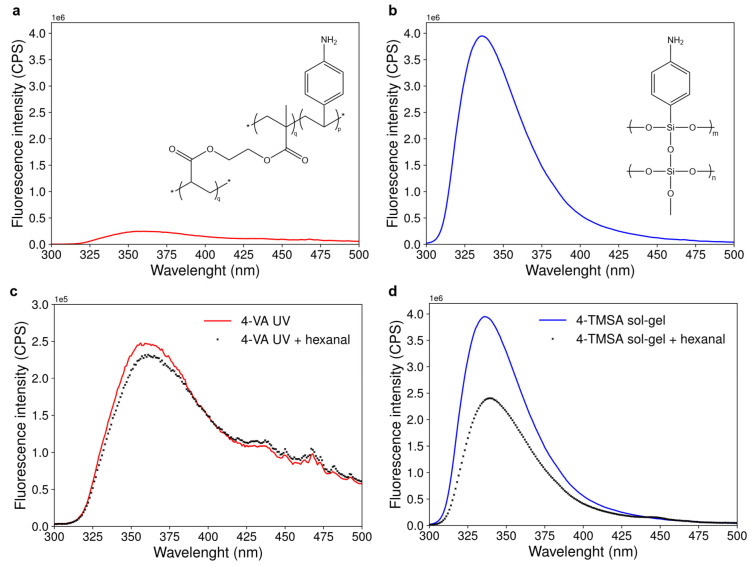
Fluorescence emission spectra of the materials and their changes upon interaction with hexanal. (**a**) Fluorescence emission spectrum of a suspension of 10 mg of the acrylate-based material in ethanol. Excitation wavelength was set at 280 ± 2 nm. p and q are the molar ratios of functional monomer and cross-linker used for the synthesis of the acrylate-based materials, respectively. (**b**) Fluorescence emission spectrum of the silicate-based material (suspension of 10 mg of material in ethanol, excitation wavelength: 290 ± 2 nm). m and n are the molar ratios of functional monomer and cross-linker used for the synthesis of the functionalized sol-gel material, respectively. (**c**) Evolution of the fluorescence emission spectrum of the acrylate-based material suspended in ethanol before and after 15 min of contact with hexanal in the liquid phase at a concentration of 9.98 × 10^−3^ mol·L^−1^. (**d**) Evolution of the fluorescence emission spectrum of the silicate-based material suspended in ethanol before and after 15 min of contact with hexanal in the liquid phase (concentration 9.98 × 10^−3^ mol·L^−1^).

As shown in Figure 3a,b, displaying the fluorescence emission spectra of the acrylate-based and the silicate-based materials, both materials were fluorescent in the UV region. The maximum of fluorescence intensity was at 360 nm and 337 nm for the acrylate-based and the silicate-based materials, respectively. The silicate-based material was 25 times more fluorescent compared to its acrylate counterpart, based on maximum fluorescence intensities. The corresponding reference materials, synthesized following the same experimental procedure but without any monomer, did not exhibit fluorescence. Therefore, only the functional monomers (4-vinylaniline for the acrylate-based material and 4-trimethoxysilylaniline for the silicate-based material) were responsible for the fluorescence of the materials.

Figure 3c,d compare the fluorescence emission spectra of the acrylate-based and the silicate-based materials before and after interaction with hexanal in the liquid phase. In both cases, a decrease in the fluorescence intensity of the material upon interaction with hexanal was observed. The percentage of fluorescence quenching by hexanal was 3.5 times more important for the silicate-based material compared to the acrylate-based material (39.5% and 11.5% of fluorescence quenching, respectively).

Both acrylate-based and silicate-based materials were thus fluorescent and showed significant changes in their fluorescence intensity after interaction with hexanal. These two materials could thus have been considered for carbonyl sensing. However, as the fluorescence of the material is quenched by carbonyls, the material with the highest fluorescence intensity will exhibit the highest range of detection and the lowest limit of detection. These are two important features for a sensor. Therefore, the silicate-based material was unambiguously the best suited for the targeted application.

### 3.5. Special Case of Materials with a Higher Ratio of Functional Monomer

**Figure 4:**
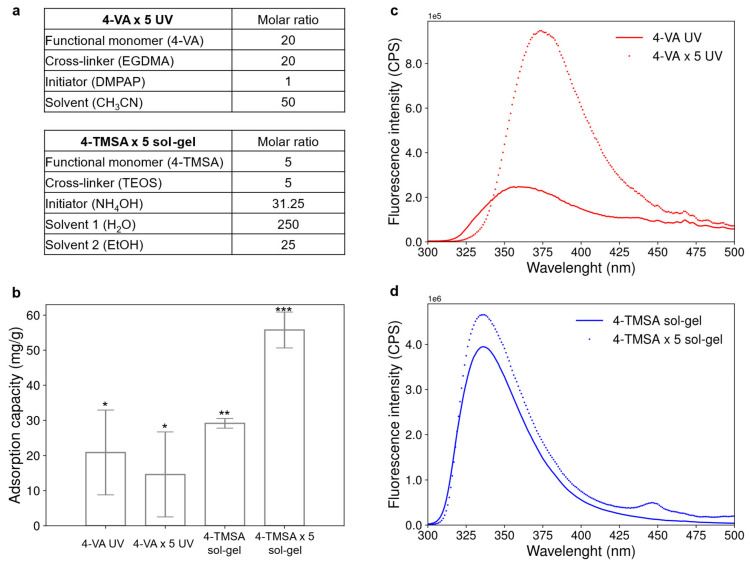
Adsorption capacity and fluorescence property of acrylate-based and silicate-based materials synthesized with a higher ratio of functional monomer. (**a**) Molar ratios used to synthesize the acrylate-based and silicate-based material with higher amount of functional monomer. (**b**) Adsorption capacity of the acrylate-based and silicate-based materials synthesized with functional monomer/cross-linker molar ratio of 1/5 or 5/5. Significantly different values (*p* < 0.05) are indicated by different number of stars. Error bars represent the standard deviation obtained for three replicates. (**c**) Fluorescence emission spectrum of the acrylate-based material initially synthesized (4-VA UV) and its analogue synthesized with five times more functional monomer (4-VA × 5 UV). (**d**) Fluorescence emission spectrum of the silicate-based material initially synthesized (4-TMSA sol-gel) and its analogue synthesized with five times more functional monomer (4-TMSA × 5 sol-gel).

To further enhance the adsorption capacity and the fluorescence of the materials, the effect of one final parameter was investigated. The two best candidates, the 4-VA UV and the 4-TMSA sol-gel materials, were synthesized under previously optimized conditions, but with a fivefold increase in the amount of functional monomer (see Figure 4a).

Figure 4b compares the adsorption capacity of the acrylate-based and silicate-based materials synthesized with a functional monomer/cross-linker molar ratio of 1/5 or 5/5. The adsorption capacity of the acrylate-based material did not significantly change with the increase in the quantity of functional monomer. However, the adsorption capacity of the silicate-based material was almost doubled when the amount of functional monomer was multiplied by five.

Figure 4c,d display the influence of the increase in functional monomer amount on the fluorescence emission spectra of the acrylate-based and silicate-based materials. The fluorescence of the materials containing five times more functional monomer was enhanced in both cases. The fluorescence intensity was multiplied by 3 and 1.2 for the acrylate-based and the silicate-based material, respectively. For the acrylate-based materials, a fivefold increase in the amount of functional monomer resulted in a shift in emission wavelength from 355 nm to 375 nm. This bathochromic shift may be attributed to the stabilization of the excited state due to the formation of aggregates involving stacked aniline rings [18]. In the case of the silicate-based materials, a fivefold increase in the amount of functional monomer led to the appearance of an additional emission peak at 448 nm. This could be attributed to the emission of aggregates formed via π-stacking interactions between aniline rings [19]. Similar to the initial materials 4-VA UV and 4-TMSA sol-gel, the fluorescence of the two materials synthesized with a higher amount of functional monomer was quenched upon interaction with hexanal (see Figure S1 in Supporting Information).

To sum up, the increase in the quantity of functional monomer during the synthesis had a strong impact on both the adsorption capacity and the fluorescence property of the silicate-based material, and only on the fluorescence properties of the acrylate-based material. However, whatever the material was (acrylate-based or silicate-based), neither the adsorption capacity nor the fluorescence intensity was increased by a factor of five, as could have been expected by multiplying by five the amount of functional monomer for the synthesis. Therefore, increasing the amount of functional monomer did not enhance the adsorption capacity or the fluorescence intensity to the expected level. To really increase the detection capacity of the material, both quantities of initiator and cross-linker should be optimized at the same time. However, the synthesis cost would consequently increase, leading to the need to find a compromise between improving the materials’ properties and the cost of the synthesis.

### 3.6. Global Overall Comparison of the Acrylate-Based and Silicate-Based Materials

**Figure 5:**
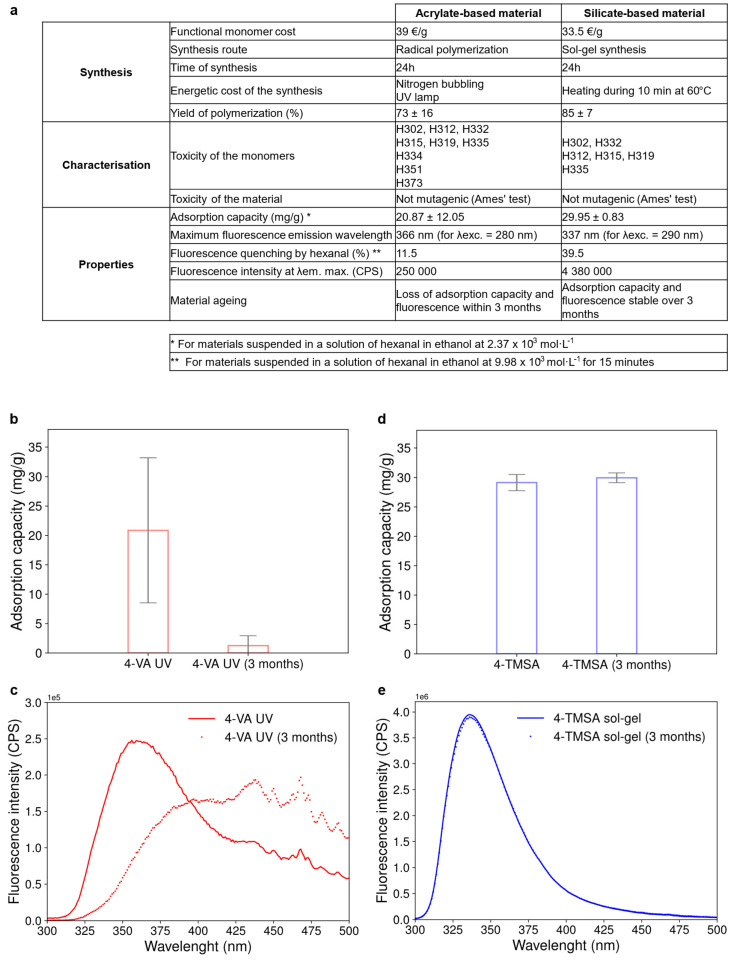
Global comparison of the acrylate-based and silicate-based materials. (**a**) Table comparing the acrylate-based and the silicate-based material regarding their synthesis, characterization and properties. (**b**) Evolution of the absorption capacity of the 4-VA UV material over three months. Error bars represent the standard deviation obtained from three replicates. (**c**) Evolution of the fluorescence emission spectrum of the 4-VA UV material over three months. (**d**) Evolution of the absorption capacity of the 4-TMSA sol-gel material over three months. Error bars represent the standard deviation obtained from three replicates. (**e**) Evolution of the fluorescence emission spectrum of the 4-TMSA sol-gel material over three months.

In a nutshell, the two types of materials—acrylate-based and silicate-based—fit the objective of developing a new material for carbonyl sensing using fluorescence. Both 4-VA UV and 4-TMSA sol-gel materials showed good adsorption capacity for hexanal as well as a subsequent change in the fluorescence property upon interaction with hexanal. To sum up, these two materials were finally compared on specific points considering their synthesis, toxicityget and adsorption capacity as well as fluorescence property (see table in Figure 5a).

Firstly, regarding the synthesis, both functional monomers (4-vinylaniline and 4-trimethoxysilylaniline) have a rather similar cost. However, acrylate-based materials are more widely used. An extensive choice in terms of monomers and synthesis conditions can be investigated. On the contrary, sol-gel polymerization is a less explored synthesis route resulting in fewer commercial functional monomers. The acrylate path thus gives more opportunities for potential further improvements of the material’s structure. However, based on the yield of polymerization results, the reproducibility of the synthesis was better controlled with the sol-gel process compared to the radical polymerization.

Secondly, solvents (ethanol and water) and reactants employed for the sol-gel synthesis (4-TMSA monomer and TEOS) are less toxic compared to those used for the synthesis of acrylate-based materials (especially 4-VA monomer, EGDMA and acetonitrile) [9]. In this example, the acrylate functional monomer may cause allergic reactions if inhaled, is suspected of causing cancer and may cause damage to organs depending on the exposure, while this is not the case for the silica functional monomer. However, both materials were not mutagenic for bacteria according to the Ames’ test. The toxicity of the monomers was thus reduced once the covalent bonds were created with the crosslinker during polymerization.

Thirdly, in terms of functional properties for the desired application, the acrylate-based material seemed less performant compared to its silicate-based counterpart. On the first hand, the acrylate-based material showed lower adsorption capacity with higher variability. On the second hand, it exhibited by far a lower fluorescence intensity and thus a lower percentage of fluorescence quenching upon interaction with hexanal. Moreover, the acrylate-based material lost its adsorption capacity and fluorescence properties within three months (see Figure 5b,c) while these characteristics remained stable over the same time for the silicate-based material (see Figure 5d,e). The material internal structure of the acrylate-based material may have collapsed with time, rendering the adsorption sites inaccessible to hexanal molecules. However, the modification of the fluorescence spectrum of the acrylate-based material and the shift in its maximum emission wavelength, as presented in Figure 5c, also suggest changes in the near environment of fluorescent aniline groups or chemical transformations of the anilines. Such aging of the material was therefore prohibitive for the use of the 4-VA UV material.

Finally, the sol-gel material seemed thus the most appropriate for the targeted application of carbonyl sensing using fluorescence.

### 3.7. Characterisation of the Materials

To better understand the differences in the performance of the materials, especially the acrylate-based ones, their chemical composition as well as their morphology were compared.

**Figure 6:**
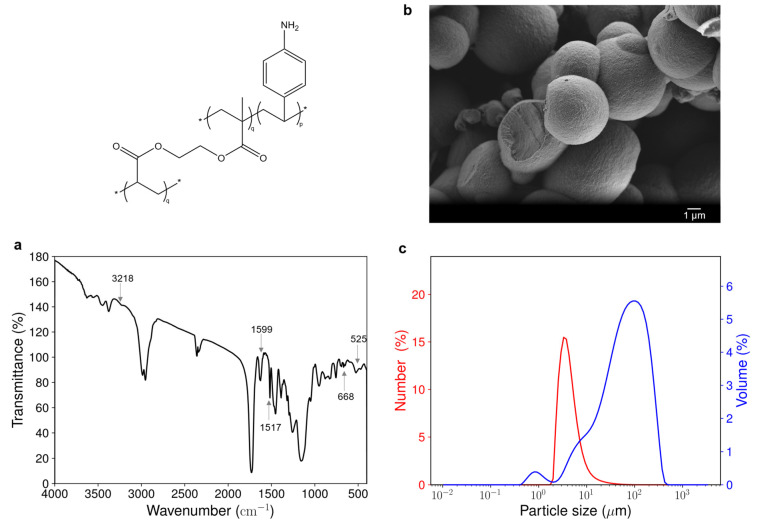
Characterization of the acrylate-based material. (**a**) FTIR absorbance spectrum of the material. The absorbance peaks represent (amine (3218 cm^−1^, 1599 cm^−1^) and aromatic ring (1517 cm^−1^, 668 cm^−1^, 525 cm^−1^). (**b**) SEM image of the material showing aggregates of spherical particles (magnification 5000). (**c**) Particle size distribution of the material obtained by laser diffraction. p and q are the molar ratios of functional monomer and cross-linker used for the synthesis of the acrylate-based materials, respectively.

Figure 6a shows the Fourier-transform infrared (FTIR) spectrum of the acrylate-based material. ν_el_ (C=C) at 1517 cm^−1^ corresponds to elongation vibrations of the aromatic ring of the aniline group. The peak at 668 cm^−1^ is attributed to an out-of-plane deformation of the (C=C-H) bonds of the aromatic ring and the one at 525 cm^−1^ is attributed to an out-of-plane aromatic ring deformation vibration of a 1,4-disubstituted benzene. These peaks clearly differentiate the spectrum of the material from that of its reference, obtained by polymerization of EGDMA only (see Figure S2a in Supporting Information). This proves the successful incorporation of the aromatic ring of the 4-vinylaniline functional monomer into the polymer material. Less intense peaks ν_sym_ (N-H) at 3218 cm^−1^ and δ_s_ (N-H) at 1599 cm^−1^ suggest the presence of primary and aromatic amine. However, the resolution of the spectrum did not allow for determining if some secondary amines appeared during polymerization, which would have suggested the participation of the aniline group in the radical polymerization. The presence of water in the material can be noted by the presence of the following characteristic peaks: ν_sym_ (O-H) at 3374 cm^−1^ and δ (H-O-H) at 1625 cm^−1^.

The morphology of the 4-VA UV material, as revealed by scanning electron microscopy (SEM), shows aggregates of spherical particles, having sizes in the micrometric range (see Figure 6b). The shape of the particle is consistent with the type of polymerization used, as spherical particles are often obtained with precipitation polymerization [20]. Particles are better defined and less agglomerated in the material compared to the reference material (see Figure S2b in Supporting Information), suggesting a higher level of structuration of the material due to the addition of the functional monomer.

This is in good agreement with quantitative analysis performed by laser light diffraction, which also shows that the 4-VA UV material has a particle size in the micrometric range (Figure 6c). The bimodal volumetric distribution indeed revealed the presence of two populations having a near-diameter of about 1 µm and 100 µm, respectively. However, the number distribution indicated that most of the particles have a size of around 4 µm. The reference material exhibited a similar particle size distribution (see Figure S2c in Supporting Information). Most particles of the reference material also have a size of around 4 µm. However, the volumetric distribution reveals the presence of bigger aggregates having sizes of about 10 µm and 400 µm. This suggests a 10-fold shrinkage of the volume of the aggregates when the functional monomer is included within the material structure.

4-TMSA sol-gel material was deeply characterized in a previous work [21] and showed a morphology comparable to that of its acrylate-based counterpart. FTIR analysis confirmed the successful incorporation of the 4-trimethoxysilylaniline monomer within the polymeric structure, resulting in a material made of irregular-shaped particles having sizes of about 1 µm and 100 µm, with most of the particles having a size around 1 µm. The important differences in terms of adsorption capacity and fluorescence property between the 4-VA UV material and the 4-TMSA sol-gel material, thus, cannot be explained by their morphological difference. Simon and Spivak suggested that the 4-vinylaniline functional monomer could inhibit the radical polymerization process [22]. This would result in a reduced amount of aniline available in the final material, which is responsible for both hexanal adsorption and fluorescence, and could thus explain the lower performance of the acrylate-based material.

Difference between 4-VA UV and 4-VA thermal materials

No significant difference in the FTIR spectra of the acrylate-based materials obtained by UV-polymerization and by thermal initiation was noticed (see Figure S3a in Supporting Information). Thus, the difference in adsorption capacity could not be attributed to any structural difference between the two materials. SEM images of the 4-VA thermal material showed similar morphology to the 4-VA UV material, with aggregates made of spherical particles. However, more spherical particles separated from each other were observed (see Figure S3b in Supporting Information). Laser diffraction analysis revealed similar volume and number distributions for both photoinitiated and thermally initiated materials (see Figure S3c in Supporting Information). The number distribution of 4-VA thermal showed slightly smaller particles than 4-VA UV (0.6 µm and 4 µm, respectively). The volume distribution of the particles obtained from 4-VA thermal aggregates slightly differs from that of the 4-VA UV, with more aggregates of 5 µm. These small morphological differences are in favor of the hypothesis that some adsorption sites are entrapped within the bulk structure of the 4-VA thermal material, leading to a smaller quantity of hexanal adsorbed by this material.

Difference between 4-VA UV and 4-VA × 5 UV

No significant morphological difference was noted when the molar ratio of the functional monomer was increased, except that the volume distribution obtained by laser diffraction revealed the presence of bigger aggregates of 200 µm for the-VA × 5 UV material instead of 100 µm for the-VA UV material.

Difference between 4-VA UV freshly synthesized and 4-VA UV after 3 months of aging

No significant difference in the FTIR spectra of the acrylate-based materials freshly synthesized and after three months of aging was noticed (see Figure S4a in Supporting Information). It is possible that a chemical or physical degradation of the material occurred. However, the characteristic peaks attributed to the primary amine were already low in intensity compared to those from the α,β-unsaturated ester from the EGDMA crosslinker (ν(C=O) at 1723 cm^−1^, and ν(C-O) at 1282 cm^−1^ and at 1144 cm^−1^) in the freshly synthesized material, due to the ratio used for the synthesis of the material (five times more cross-linker than functional monomer). The degradation of this functional group with time was thus hardly noticeable on FTIR spectra. A collapse of the internal structure of the material could have explained the loss of absorption and fluorescence properties over time. This hypothesis was supported by laser diffraction analysis that revealed a shrinkage of the particle size over time, from 4 µm for freshly synthesized material to 0.6 µm for aged material (after three months) (see Figure S4b in Supporting Information). Aniline groups entrapped within the material structure are thus probably less accessible to hexanal for adsorption at the material surface, leading to a decrease in the adsorption capacity of the material with time. Similarly, remaining aniline groups could not be excited if they were entrapped within the polymeric structure, resulting in a decrease in the fluorescence of the material.

## 4. Conclusions

In the aim of designing a carbonyl sensor based on fluorescence, many material synthesis parameters were studied, including polymerization type, radical polymerization initiation method, the nature of the functional monomer and the molar ratio of the different reactants. They significantly impacted the performance of the materials in terms of yield of polymerization, adsorption capacity and fluorescence properties. It was shown that a reduced solvent volume favored material precipitation. Materials based on 4-vinylaniline monomer showed a better adsorption capacity compared to those based on 3-vinylaniline and 2-vinylaniline. Materials obtained by UV-polymerization displayed a better adsorption capacity compared to those obtained by thermally initiated polymerization. However, compared to all acrylate-based materials tested, the silicate-based material was considered the best choice for the desired application. The good reproducibility of the synthesis, its higher adsorption capacity and its higher fluorescence intensity were important parameters that supported such selection. The stability of its adsorption capacity and fluorescence over time was also decisive.

## Figures and Tables

**Table 1:** Names of the different synthesized materials, based on the main selected parameters for optimizing their synthesis.

Name of the Material	Polymerization Type	Initiation of the Polymerization	Nature of Functional Monomer	Functional Monomer/Cross-Linker Ratio
4-VA UV	Radical polymerization	UV radiation	4-VA	4/20
3-VA UV	Radical polymerization	UV radiation	3-VA	4/20
2-VA UV	Radical polymerization	UV radiation	2-VA	4/20
4-VA thermal	Radical polymerization	Heat	4-VA	4/20
3-VA thermal	Radical polymerization	Heat	3-VA	4/20
2-VA thermal	Radical polymerization	Heat	2-VA	4/20
4-TMSA sol-gel	Sol-gel polymerization	n.a.	4-TMSA	1/5
4-VA × 5 UV	Radical polymerization	UV radiation	4-VA	20/20
4-TMSA × 5 sol-gel	Sol-gel polymerization	n.a.	4-TMSA	5/5

4-VA UV stands for the material synthesized by photo-initiated radical polymerization using 4-vinylaniline monomer. 4-VA thermal refers to the material synthesized by radical polymerization thermally initiated using 4-vinylaniline monomer. 3-VA and 2-VA are used instead of 3-vinylaniline and 2-vinylaniline, respectively. 4-TMSA sol-gel indicates that the material was synthesized by the sol-gel process using 4-trimethoxysilylaniline monomer. n.a. means not applicable.

## Data Availability

The original contributions presented in this study are included in the article/Supplementary Material. Further inquiries can be directed to the corresponding author(s).

## Supplementary Materials

The following supporting information can be downloaded at https://www.mdpi.com/article/10.3390/polym17131843/s1, Figure S1: Evolution of the fluorescence emission spectrum of the acrylate-based and silicate-based 9 material synthesized with higher ratio of functional monomer in presence of hexanal; Figure S2: Chemical and physical characterization of the reference material; Figure S3: Chemical and physical characterization of the acrylate-based material synthesized by radical polymerization thermally initiated; Figure S4: Chemical and physical characterization of the acrylate-based material three months old.
