# Supplementary material for: Design and Synthesis Optimization of Fluorescent Acrylate-Based and Silicate-Based Materials for Carbonyl Adsorption
Source: Polymers (Basel). 2025 Jun 30;17(13):1843. doi: 10.3390/polym17131843 (PMC12252228; doi:10.3390/polym17131843)
Supplement: Supplementary file 1 [file polymers-17-01843-s001.zip › polymers-3686710-supplementary.pdf]

SUPPORTING INFORMATION

Design and synthesis optimization of acrylate-based and silicate-based materials for carbonyl sensing based on fluorescence.

Laura Carballido[a], Thomas Karbowiak[a], Elias Bou-Maroun [a]**

[a] Université Bourgogne Europe, Institut Agro, INRAE, UMR PAM, F-21000 Dijon, France

* Corresponding authors:

elias.bou-maroun@agrosupdijon.fr

thomas.karbowiak@institut-agro.fr

Keywords: acrylates, sol-gel, fluorescence, sensor, lipid oxidation

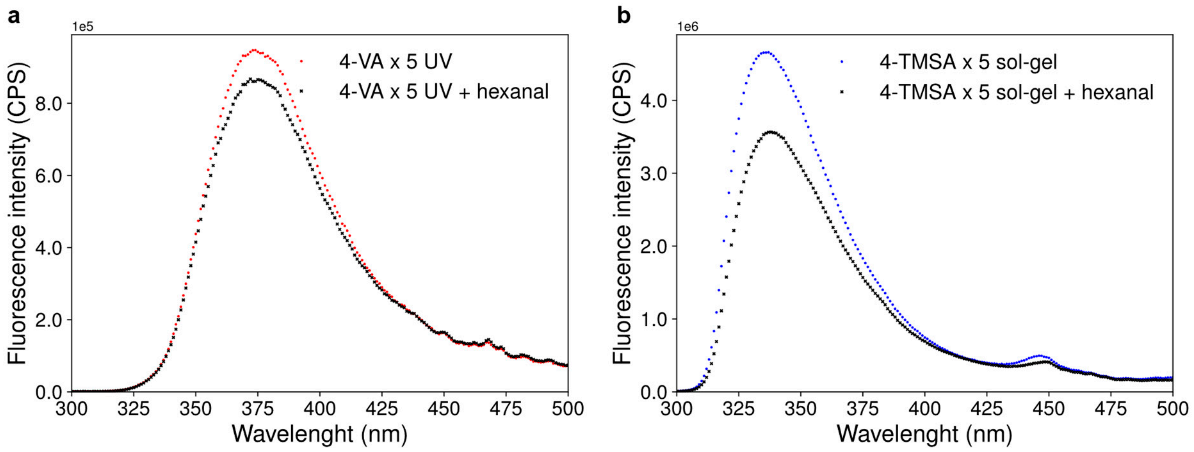

Figure S1: Evolution of the fluorescence emission spectrum of the acrylate-based and silicate-based material synthesized with higher ratio of functional monomer in presence of hexanal.

a. Evolution of the fluorescence emission spectrum of the 4-VA x 5 UV material suspended in ethanol before and after 15 minutes of contact with hexanal in the liquid phase (concentration 9.98 x 10^{-3} mol·L^{-1}).

b. Evolution of the fluorescence emission spectrum of the 4-TMSA x 5 sol-gel material suspended in ethanol before and after 15 minutes of contact with hexanal in the liquid phase (concentration 9.98 x 10^{-3} mol·L^{-1}).

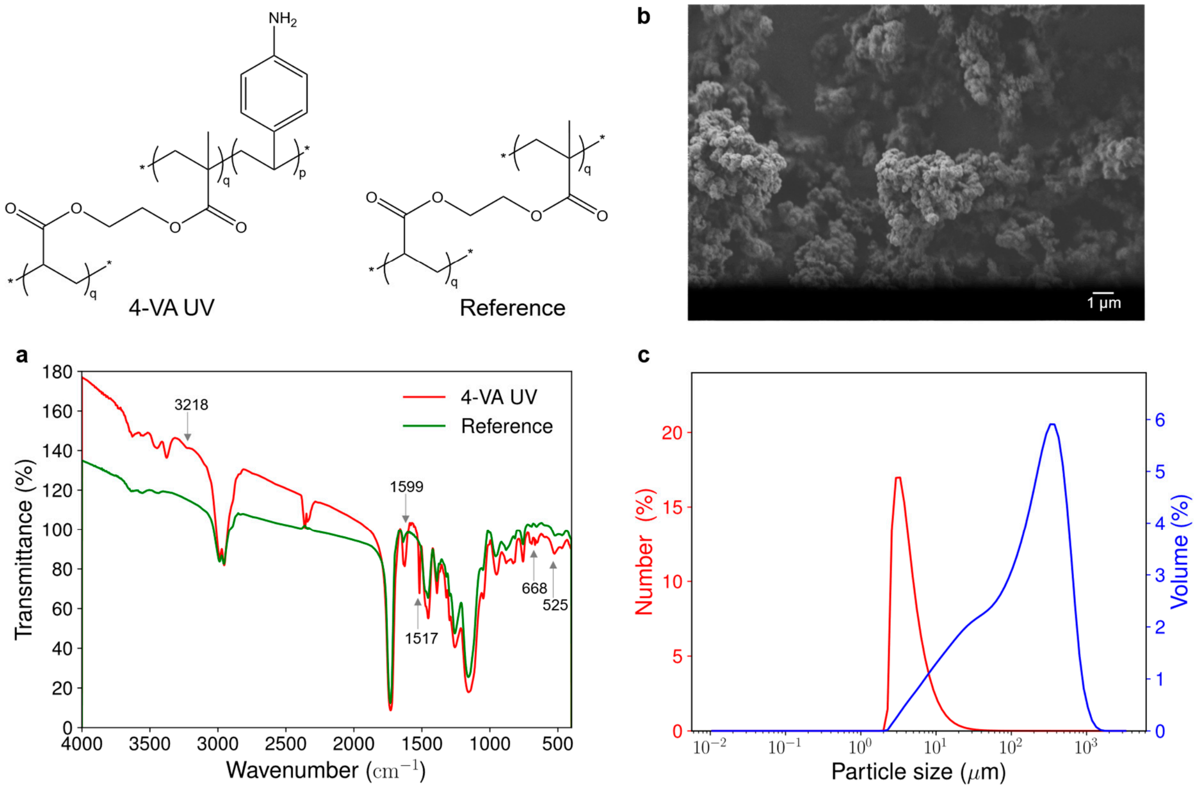

Figure S2: Chemical and physical characterization of the reference material.

a. Compared FTIR absorbance spectra of the 4-VA UV material and its reference. Characteristic peaks (amine (3218 cm^{-1}, 1599 cm^{-1}) and aromatic ring (1517 cm^{-1}, 668 cm^{-1}, 525 cm^{-1}) in the 4-VA UV material differentiate the two spectra, suggesting the successful incorporation of the functional monomer into the material.

b. SEM image (magnification 5000) of the reference material showing aggregates of spherical particles less defined and more agglomerated compared to the 4-VA UV material.

c. Particle size distribution of the reference material obtained by laser diffraction. The bimodal volumetric distribution reveals the presence of two populations having sizes of about 1 µm and 400 µm. The number distribution indicates that most of the particles have a size around 4 µm.

p and q are the molar ratio of functional monomer and cross-linker used for the synthesis of the acrylate-based materials, respectively.

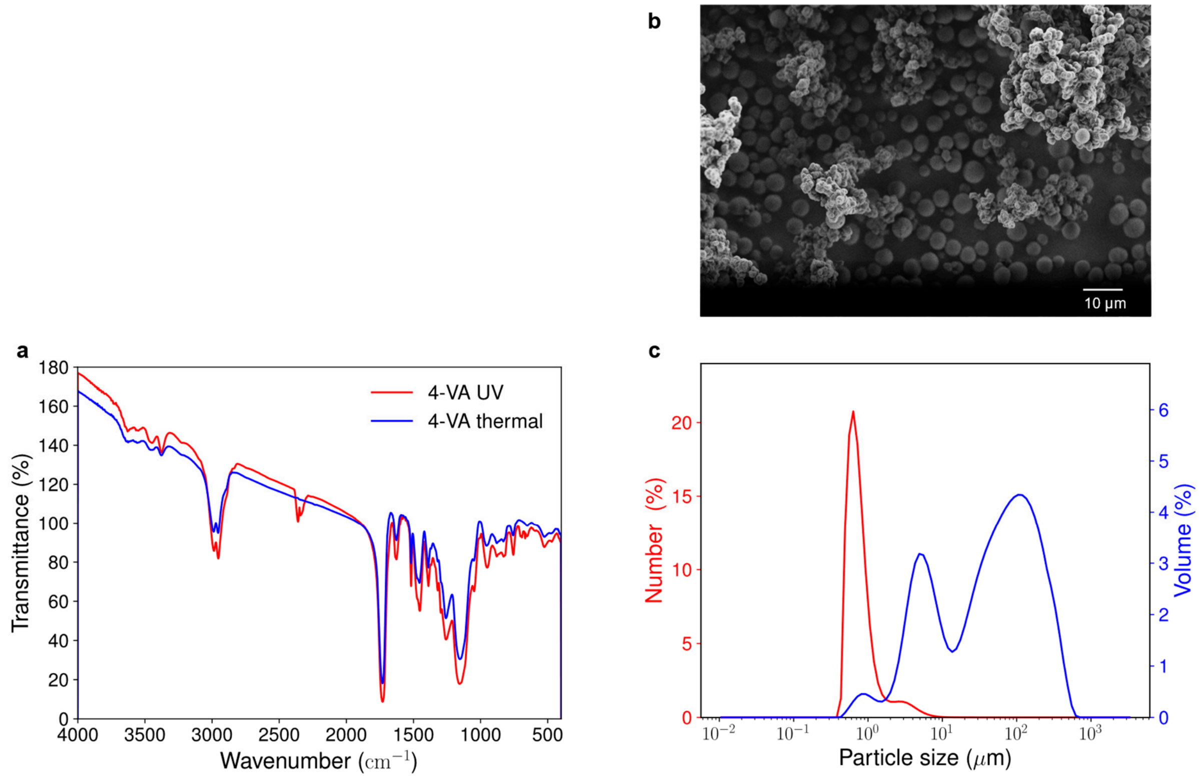

Figure S3: Chemical and physical characterization of the acrylate-based material synthesized by radical polymerization thermally initiated.

a. Compared FTIR absorbance spectra of the 4-VA UV and 4-VA thermal material, showing no significant differences.

b. SEM image (magnification 1000) of the 4-VA thermal material showing aggregates of spherical particles with more stand-alone particles compared to the 4-VA UV material.

c. Particle size distribution of the 4-VA thermal material obtained by laser diffraction. The volumetric distribution reveals the presence of three populations having sizes of about 1 µm, 5 µm and 100 µm. The number distribution indicates that most of the particles have a size around 0.6 µm.

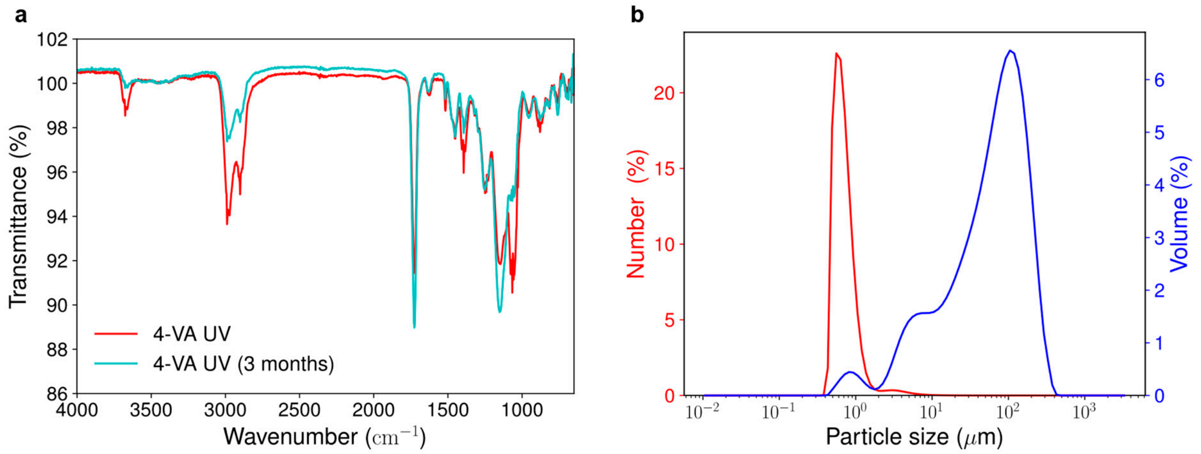

Figure S4: Chemical and physical characterization of the acrylate-based material three months old.

a. *Compared FTIR absorbance spectra of the 4-VA UV freshly synthesized and 4-VA UV three months old material, showing no significant differences. The resolution of the spectra may not be good enough to distinguish small changes.*

b. *Particle size distribution of the three months old material obtained by laser diffraction. The volumetric distribution reveals the presence of three populations having sizes of about 1 µm, 5 µm and 100 µm. The number distribution indicates that most of the particles have a size around 0.6 µm. A 6-fold shrinkage of the particle size is observed, suggesting a collapse of the structure of the material with time.*
